# Recruitment of lysine demethylase 2A to DNA double strand breaks and its interaction with 53BP1 ensures genome stability

**DOI:** 10.18632/oncotarget.24636

**Published:** 2018-03-23

**Authors:** Murilo T.D. Bueno, Marta Baldascini, Stéphane Richard, Noel F. Lowndes

**Affiliations:** ^1^ Segal Cancer Center, Sir Mortimer B Davis Jewish General Hospital, Lady Davis Institute for Medical Research, Montréal, Québec, Canada; ^2^ Department of Medicine, McGill University, Montréal, Québec, Canada; ^3^ Department of Oncology, McGill University, Montréal, Québec, Canada; ^4^ Genome Stability Laboratory, Centre for Chromosome Biology, National University of Ireland, Galway, Ireland

**Keywords:** DNA double strand break repair, genome stability, KDM2A, 53BP1, ubiquitin

## Abstract

Lysine demethylase 2A (KDM2A) functions in transcription as a demethylase of lysine 36 on histone H3. Herein, we characterise a role for KDM2A in the DNA damage response in which KDM2A stimulates conjugation of ubiquitin to 53BP1. Impaired KDM2A-mediated ubiquitination negatively affects the recruitment of 53BP1 to DSBs. Notably, we show that KDM2A itself is recruited to DSBs in a process that depends on its demethylase activity and zinc finger domain. Moreover, we show that KDM2A plays an important role in ensuring genomic stability upon DNA damage. Depletion of KDM2A or disruption of its zinc finger domain results in the accumulation of micronuclei following ionizing radiation (IR) treatment. In addition, IR-treated cells depleted of KDM2A display premature exit from the G2/M checkpoint. Interestingly, loss of the zinc finger domain also resulted in 53BP1 focal distribution in condensed mitotic chromosomes. Overall, our data indicates that KDM2A plays an important role in modulating the recruitment of 53BP1 to DNA breaks and is crucial for the preservation of genome integrity following DNA damage.

## INTRODUCTION

Lysine demethylase 2A (KDM2A), also known as JHDM1A/FBXL11, was the first Jumonji C (JmjC)-domain-containing histone demethylase to be identified [1]. JmjC domains are present in metalloenzymes that function in histone demethylation and characterize the Jumonji family of transcription factors [2]. KDM2A has been implicated in the regulation of gene transcription [1, 3] by preferentially demethylating Lys36 on histone H3 (H3K36me2), a histone mark commonly associated with gene activation [4]. KDM2A also contains a CXXC-type zinc finger domain, a PHD domain, one F-box domain and numerous C-terminal leucine-rich repeats. Both over and under expression of KDM2A have been linked to oncogenesis [5] and, although the underlying mechanisms have not been defined, depletion of KDM2A has also been linked to cell survival upon genotoxic damage [3, 6].

The p53-binding protein 1 (53BP1) is a major regulator of DNA double-strand break (DSB) repair [7]. In particular, by impeding resection, 53BP1 preferentially promotes the non-homologous end-joining DNA repair pathway [7]. 53BP1 specifically localizes to DSBs and its recruitment to damaged chromatin is essential for maintenance of genomic integrity [7]. The accumulation of 53BP1 at DSBs relies on the recognition of ubiquitinated Lys15 on histone 2A (H2AK15ub) and dimethylated Lys20 on histone 4 (H4K20me2)[8, 9]. These histone marks are recognized by 53BP1 through its tandem tudor domain [9] and ubiquitylation-dependent recruitment (UDR) motif, respectively.

The localization of 53BP1 to DSBs is directly dependent on the ubiquitination of chromatin that surrounds DNA breaks. The E3 ubiquitin ligases RNF8 and RNF168 play a major role in this process [7, 10, 11]. In fact, RNF8-dependent and K63-linked ubiquitination of the linker histone H1 at DSBs [12], and possibly other substrates, provides a high affinity docking site for RNF168 which in turn is responsible for ubiquitination of H2AK15 and subsequent 53BP1 UDR-dependent recruitment [13]. Furthermore, RAD18 and BRCA1 have similarly been shown to interact with 53BP1 and promote its ubiquitination [14, 15].

More recently, KDM2A and its closest homolog, KDM2B/FBXL10, have both been reported to promote ubiquitination of β-catenin and c-Fos, respectively [16, 17]. The levels of KDM2A-induced ubiquitination of β-catenin were shown to depend on the demethylase activity of KDM2A. In addition, both JmjC and PHD domains of KDM2A were necessary to promote β-catenin degradation [16]. KDM2B was reported to form an SCF (SKP1-CUL1-F-box) E3 ubiquitin ligase complex via the interaction between its F-box domain and SKP1/CUL1. Moreover, the C-terminal leucine-rich repeats of KDM2B were shown to mediate the binding between KDM2B and its substrate c-Fos [17]. It remains unknown whether KDM2A functions in an SCF E3 ubiquitin ligase complex. These findings indicate that ubiquitination by KDM2-family of lysine demethylases exerts significant cellular functions.

In this report, we demonstrate that KDM2A associates with and ubiquitinates 53BP1. Impaired interaction between these two proteins negatively affected 53BP1 recruitment to DSBs and resulted in increased DNA damage-induced genomic instability. Moreover, we provide data showing that KDM2A accumulates at DSBs via a mechanism that depends on its demethylase activity and zinc finger domain. We also report the occurrence of DNA damage-stimulated 53BP1focal accumulation in mitotic cells expressing a ubiquitination-defective mutant of KDM2A.

## RESULTS

### KDM2A interacts with 53BP1

**Figure 1:**
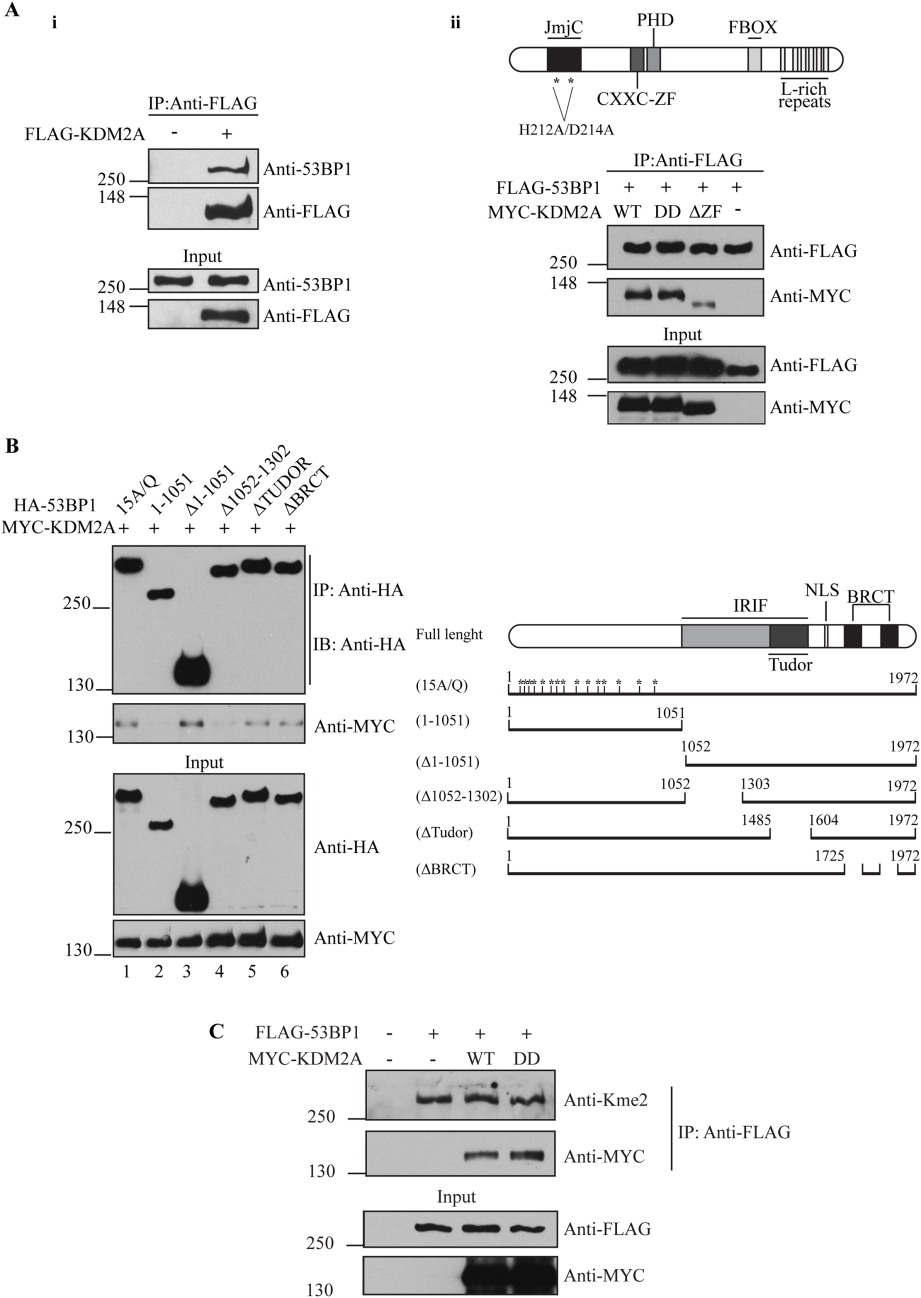
KDM2A associates with 53BP1 **(A-i)** KDM2A interacts with endogenous 53BP1. HEK293T extracts from cells transfected with an empty vector or FLAG-tagged KDM2A were subjected to immunoprecipitation with anti-FLAG beads. Interaction between KDM2A and 53BP1 was observed by immunoblotting with anti-53BP1. **(A-ii)** Deletion of the zinc finger domain (564-610) of KDM2A disrupts its interaction with 53BP1. Schematic of KDM2A protein structure (top panel). FLAG-tagged 53BP1 co-expressed with wild type MYC-tagged KDM2A (WT), demethylase-deficient KDM2A (DD) or KDM2A lacking the zinc finger domain (ΔZF) was immunoprecipitated with anti-FLAG beads and immunoblotted with anti-MYC and anti-FLAG. **(B)** The region comprising residues 1052-1302 of 53BP1 is necessary for its interaction with KDM2A. Schematic of full lenght 53BP1 and mutants (right panel). Lysates from cells expressing HA-tagged 53BP1 mutants and MYC-KDM2A-WT were immunoprecipitated with anti-HA and immunoblotted with anti-HA and anti-MYC. **(C)** 53BP1 harbors methylated lysine residues. Lysates from cells expressing FLAG-53BP1, co-expressing or not MYC-tagged KDM2A variants, were immunoprecipitated with anti-FLAG beads and immunoblotted with anti-MYC, anti-FLAG and pan-dimethyl lysine antibody (anti-Kme2).

53BP1 has been identified as a potential KDM2A interactor by proteomic studies [18, 19]. Moreover, KDM2A was demonstrated to associate with ATM [20, 45], previously shown to interact and phosphorylate 53BP1 [21–23]. To independently validate whether KDM2A associates with 53BP1, we immunoprecipitated wild-type FLAG-tagged KDM2A from HEK293T cells and probed immunoprecipitates with anti-53BP1 (Figure 1Ai). Indeed, we observed that endogenous 53BP1 co-precipitated with FLAG-KDM2A.

RAD18 was shown to interact with 53BP1 via its zinc finger domain [14]. Thus, we evaluated whether the zinc finger domain of KDM2A also mediates the interaction between this protein and 53BP1. In fact, immunoprecipitation analyses demonstrated that deletion of the amino acid residues comprising the CXXC-ZF domain of KDM2A (KDM2A-ΔZF) significantly impaired the association between MYC-KDM2A and FLAG-53BP1 (Figure 1Aii). However, the lysine demethylase activity of KDM2A did not affect its interaction with 53BP1, since demethylase-deficient KDM2A-DD, harbouring mutations H212A/D214A known to abrogate demethylase activity [24, 25], efficiently associated with 53BP1.

In order to identify the region of 53BP1 necessary for its interaction with KDM2A, we analyzed a panel of previously described 53BP1 mutants [26]. Co-expression of HA-tagged 53BP1 phospho-mutant 15A/Q, which has all 15 (S/T) Q sites changed to AQ, with full length MYC-KDM2A revealed that the association between these proteins does not depend on the phosphorylation status of 53BP1 (Figure 1B, lower panel). However, analysis of a 53BP1 fragment corresponding to the N-terminal 1051 amino acids demonstrated that these amino acid residues are insufficient to support an interaction with KDM2A (Figure 1B, lower panel, compare lanes 1 and 2). In contrast, a C-terminal 53BP1 fragment lacking amino acids 1 to 1051 (Δ1-1051) associated with KDM2A, indicating that the C-terminal region of 53BP1 is necessary for this interaction (Figure 1B, lower panel, compare lanes 2 and lane 3). Further mapping revealed that amino acid residues 1052-1302 are critical for the association between 53BP1 and KDM2A (Figure 1B, lane 4), whereas neither the Tudor nor the BRCT domains are required (Figure 1B, lanes 5 and 6).

Little is known about lysine methylation within 53BP1. Only three lysine residues have been reported to undergo methylation, but the relevance of these modifications remains undetermined [27]. Since we confirmed an interaction between 53BP1 and the lysine demethylase KDM2A, we evaluated the methylation status of 53BP1 lysine residues. Given that antibodies specific for lysine methylated 53BP1 are not available, we used a pan-dimethyl lysine antibody (anti-Kme2) which has been successfully utilized to detect lysine methylation of non-histone proteins [28–30]. Lu et al. used this antibody to detect partial demethylation of β-catenin [16] promoted by KDM2A.

Interestingly, immunoprecipitation of 53BP1 followed by immunoblotting with anti-Kme2 suggested that 53BP1 harbours methylated lysine residues (Figure 1C). However, we did not observe a change in the lysine methylation status of 53BP1 when either wild type KDM2A or the demethylase-deficient KDM2A mutant (KDM2A-DD) were expressed, indicating that KDM2A does not affect the methylation of 53BP1 detected using this assay.

### KDM2A expression stimulates 53BP1 ubiquitination and modulates its stability

KDM2A has been shown to stimulate ubiquitination of β-catenin and, ultimately, regulate the stability of this protein [16]. Therefore, we next tested whether KDM2A could affect 53BP1 ubiquitination.

**Figure 2:**
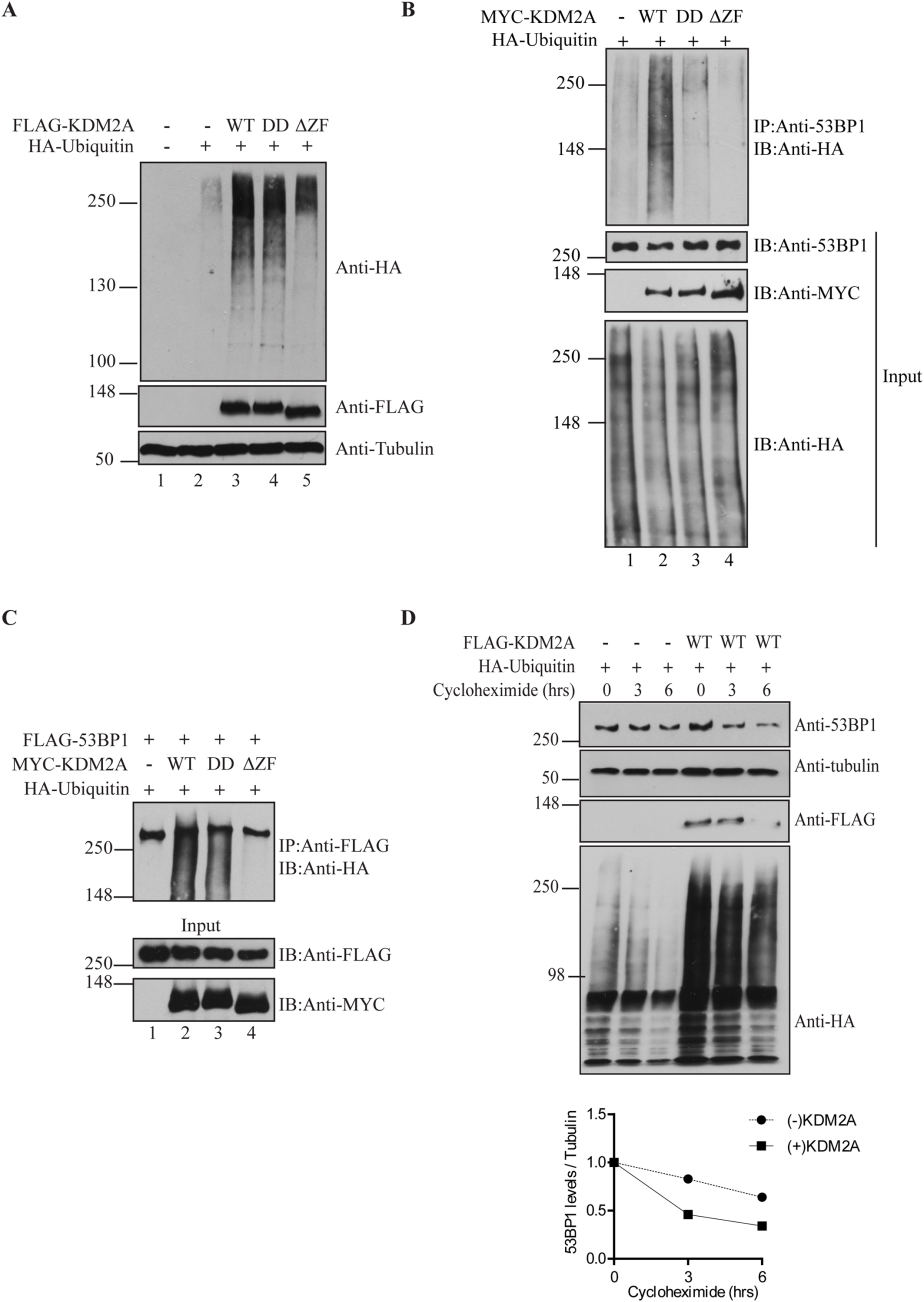
KDM2A modulates 53BP1 ubiquitination and stability **(A)** KDM2A expression enhances overall levels of protein ubiquination. Total cell lysates from HEK293T cells transfected or not with 1μg of pLPC-Puro-FLAGKDM2A-WT or KDM2A mutants, and low amount of pRK5-HA-Ubiquitin-WT (0.2μg) were immunoblotted with anti-HA, anti-FLAG and anti-Tubulin. **(B)** Stimulation of 53BP1 ubiquitination is dependent on the demethylase activity and zinc finger domain of KDM2A. Lysates from cells transfected with MYC-tagged KDM2A variants (2μg) and HA-Ubiquitin (1μg) were immunoprecipitated with anti-53BP1 and endogenous ubiquitinated 53BP1 was detected by immunoblotting with anti-HA. **(C)** Lysates from cells co-transfected with pcDNA5-FRT/T0-Flag-53BP1 (1μg), plasmids encoding for MYC-KDM2A (1μg) variants and HA-Ubiquitin (0.5μg) were immunoprecipitated with anti-FLAG beads and immunoblotted with anti-HA, anti-FLAG and anti-MYC. **(D)** KDM2A expression regulates 53BP1 protein stability. Cells transfected with HA-Ubiquitin (1μg) in the presence or absence of exogenous FLAG-KDM2AWT (2μg) were treated with cycloheximide (50μg/ml) and lysed at the indicated time points. Levels of endogenous 53BP1 were detected by immunoblotting with anti-53BP1. Additionally, immunoblotting was performed with anti-Tubulin, anti-FLAG and anti-HA. A graph displaying the ratio between 53BP1 and tubulin protein levels determined with ImageJ is shown at the bottom.

In order to estimate a potential role of KDM2A in stimulating overall protein ubiquitination, we co-expressed wild type KDM2A-WT, demethylase-deficient (KDM2A-DD) or zinc finger deletion (KDM2A-ΔZF) mutants with HA-Ubiquitin in HEK293T cells. We used low level expression of HA-Ubiquitin in order to increase the sensitivity of this assay. Immunoblotting analysis with anti-HA antibody revealed a robust increase in total protein ubiquitination in samples co-expressing HA-Ubiquitin and FLAG-KDM2A-WT or FLAG-KDM2A-DD when compared to cells expressing only HA-Ubiquitin (Figure 2A, compare lanes 2 with 3 and 4). However, expression of the zinc finger deletion mutant (KDM2A-ΔZF) displayed significantly less ubiquitination of cellular proteins detected in this assay (Figure 2A, compare lanes 3 and 4 with 5), suggesting that KDM2A-mediated enhanced ubiquitination of multiple proteins is dependent on an intact zinc finger.

Next, we conducted immunoprecipitations to assess the effect of KDM2A on 53BP1-specific ubiquitination. Compared to samples expressing only HA-Ubiquitin, the levels of ubiquitinated endogenous 53BP1 were substantially increased by co-expression of wild type KDM2A-WT (Figure 2B, compare lanes 1 and 2), while the demethylase-deficient mutant (KDM2A-DD) only marginally enhanced 53BP1 ubiquitination. Importantly, the zinc finger deletion mutant (KDM2A-ΔZF) completely failed to stimulate ubiquitination of 53BP1 (Figure 2B, compare lanes 2 and 4). Although the levels of total ubiquitinated proteins look similar in Figure 2B, cells shown in Figure 2A were transfected with 5-fold less plasmid encoding for ubiquitin to increase the sensitivity of the assay and prevent saturation of protein ubiquitination.

To further confirm these results, we performed immunoprecipitations with lysates from cells expressing exogenous FLAG-tagged 53BP1. Immunoblotting analyses confirmed the ability of wild type KDM2A to stimulate 53BP1 ubiquitination (Figure 2C, compare lanes 1 and 2). However, probably due to higher 53BP1 protein levels because of exogenous expression of 53BP1, expression of the KDM2A-DD mutant also resulted in a significant increase in 53BP1 ubiquitination. This result is unlikely due to differences in the amount of soluble and chromatin-associated 53BP1 since all immunoprecipitations were carried out with lysis buffer containing benzonase, a nuclease that digests both DNA and RNA.

Nevertheless, and in support of the previous results, the KDM2A-ΔZF mutant was incapable of stimulating ubiquitination of exogenous 53BP1 (Figure 2C, compare lanes 2 and 3 with 4). Thus, with either endogenous or overexpressed 53BP1, we observed that the zinc finger motif of KDM2A is required for 53BP1 ubiquitination.

Interestingly, the enhanced ubiquitination of 53BP1 caused by KDM2A expression seemed to result in increased degradation of 53BP1, as suggested by smeared bands observed below full-length 53BP1 (Figure 2C, lanes 2 and 3). Thus, we measured the protein stability of 53BP1 upon KDM2A expression using the protein synthesis inhibitor, cycloheximide (Figure 2D). HEK293T cells expressing HA-Ubiquitin with concomitant expression of wild type FLAG-KDM2A or empty vector, were treated with cycloheximide, lysed at the indicated time points and total cell lysates were analysed by immunoblotting. Quantification of 53BP1 levels normalized to Tubulin indicated that decreased 53BP1 stability correlated with enhanced ubiquitination by KDM2A (Figure 2D, lower panel).

### KDM2A modulates the recruitment of 53BP1 to DSBs and its depletion results in premature exit from the G2/M checkpoint induced by ionising radiation

The recruitment of 53BP1 to DNA damage sites is necessary to ensure proper DNA repair [7]. Thus, we evaluated whether KDM2A could influence the formation of 53BP1 foci upon ionizing radiation (IR).

To analyse the effects of down regulation or overexpression of KDM2A on 53BP1 foci formation, we generated U2OS cells stably expressing wild type (WT), demethylase-defective (KDM2A-DD) or zinc finger deleted (KDM2A-ΔZF) MYC-tagged KDM2A. Transfection of siRNA targeting the 3’-UTR region of the KDM2A mRNA resulted in efficient downregulation of endogenous KDM2A, when compared to cells transfected with control siRNA (Supplementary Figure 1A, compare lanes 1 and 2). We stably overexpressed MYC-KDM2A-WT, MYC-KDM2A-DD and MYC-KDM2A-ΔZF mutant in these cells as assessed by immunoblotting (Supplementary Figure 1A, lanes 3-5).

**Figure 3:**
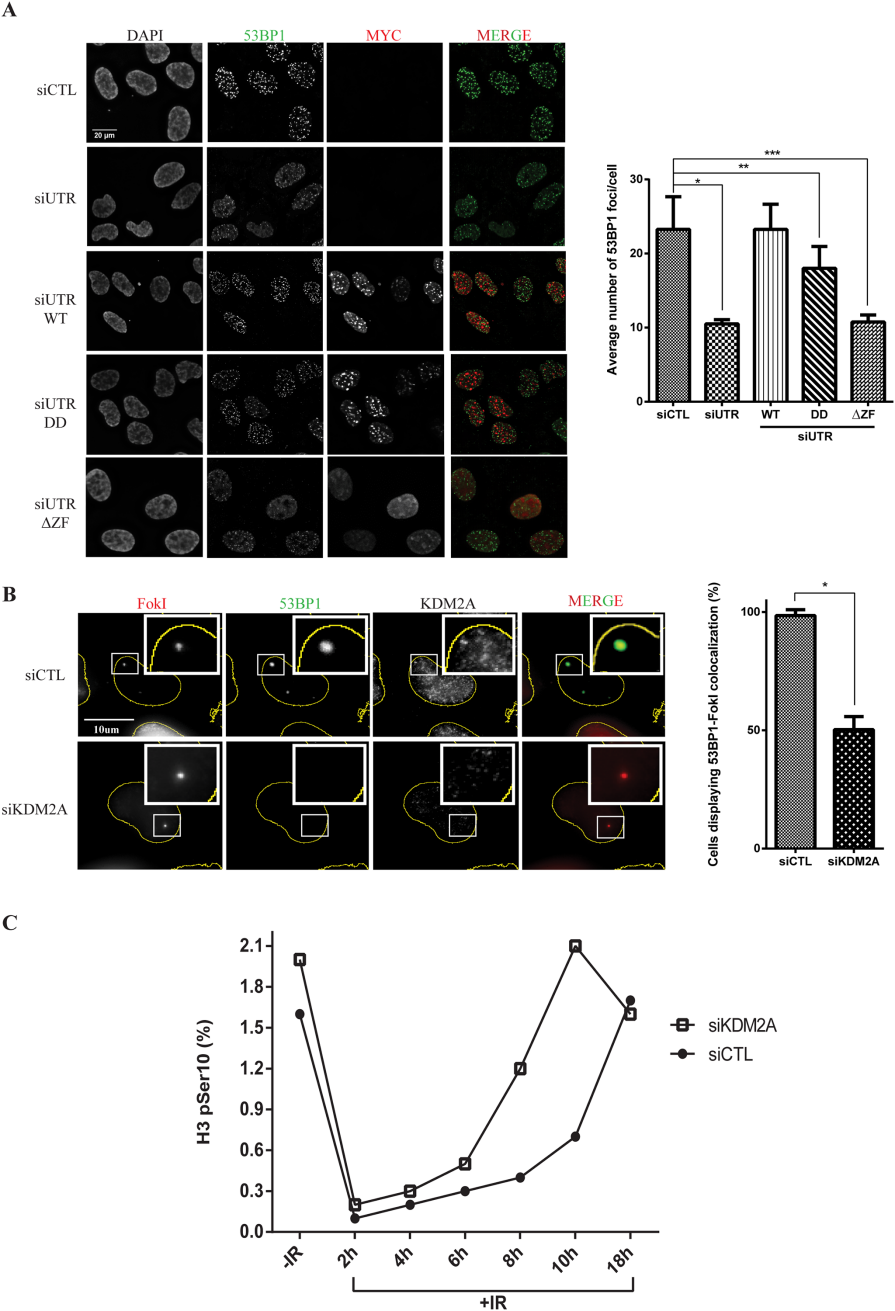
KDM2A modulates the recruitment of 53BP1 to DSBs and entry into mitosis upon IR **(A)** KDM2A modulates 53BP1 accumulation at DSBs generated by irradiation. U2OS cells transfected with control siRNA (siCTL) or siRNA targeting the 3’-UTR region of the KDM2A mRNA (siUTR) and stably expressing MYC-tagged KDM2A variants were irradiated (2Gy), fixed 1h later and subjected to immunofluorescence analysis with DAPI, anti-53BP1 (green) and anti-MYC (red). Quantification of immunofluorescence analyses (right panel) shows the average number of 53BP1 IRIF per cell in each condition. Experiments were performed in triplicate. Single (^*^), double (^**^) and triple (^***^) asterisks indicate p<0.05, two-tailed paired Student t-test. Error bars indicate standard deviation. **(B)** KDM2A depletion impairs 53BP1 recruitment to DNA breaks generated by FokI endonuclease. U2OS-DSB-reporter cells transfected with siCTL or siKDM2A were treated with Shield-1 and 4-OHT to promote expression of mCherry-LacI-FokI (FokI) (red). Immunofluorescence analyses were conducted with anti-53BP1(green) and anti-KDM2A. Quantification of immunofluorescence analyses (right panel) shows the percentage of cells displaying colocalization between 53BP1 and FokI. Asterisk indicates p<0.05, two-tailed paired Student t-test. Error bars indicate standard deviation. **(C)** KDM2A depletion results in faster progression into mitosis without proper repair of damaged DNA. U2OS cells transfected with siCTL or siKDM2A were left untreated or irradiated (2Gy) and harvested at the indicated time points after irradiation. The percentage of mitotic cells, as indicated by H3pSer10 staining, was determined by flow cytometry. Data shown is representative of experiments replicated three times.

Immunofluorescence analyses showed that depletion of endogenous KDM2A resulted in a significant reduction in 53BP1 IRIF detected in cells 1 hour after treatment with 2Gy IR (Figure 3A). Importantly, this defective recruitment of 53BP1 to DSBs caused by depletion of endogenous KDM2A could be reversed by concomitant expression of MYC-KDM2A-WT. However, cells expressing the demethylase-defective (MYC-KDM2A-DD) and, in particular, the zinc finger deleted (MYC-KDM2A-ΔZF) mutant forms of KDM2A, displayed significantly less 53BP1 IRIF.

As shown in Figure 2B, while both demethylase and zinc finger domains of KDM2A play a role in stimulating ubiquitination of endogenous 53BP1, deletion of the zinc finger domain resulted in a more severe ubiquitination defect. Thus, our data shows that deficient conjugation of ubiquitin to 53BP1 in the presence of these mutants is consistent with a reduction in the focal recruitment of 53BP1 to sites of DNA damage. In addition, we evaluated whether KDM2A depletion impacted the overall formation of IRIF by proteins harbouring ubiquitin chains. Immunofluorescence analyses using an anti-FK2 antibody (specific to ubiquitin chains), revealed that irradiated KDM2A deficient cells displayed similar FK2 IRIF when compared to cells transfected with control siRNA (Supplementary Figure 2). To further support these observations, we used a previously described U2OS reporter cell line, U2OS-DSB-reporter [31, 32], that stably expresses a fusion protein termed, ER-mCherry-LacI-FokI-DD, which is composed of a modified estradiol receptor fused to mCherry, a lac repressor protein (LacI), the nuclease domain of the FokI endonuclease and a destabilization domain (DD). Upon treatment with the Shield-1 ligand and 4-hydroxytamoxifen (4-OHT), ER-mCherry-LacI-FokI-DD is stabilized, re-localised to the nucleus and directed via its LacI moiety to bind to an integrated array of 256 LacO repeats at a single genomic locus. The FokI endonuclease moiety then generates numerous DNA double strand breaks at these LacO repeats. The localization of ER-mCherry-LacI-FokI-DD can be directly visualized via its mCherry moiety (Supplementary Figure 1B).

As expected, immunofluorescence analyses of U2OS-DSB-reporter cells treated with Shield-1 and 4-OHT and pre-extracted with cytoskeletal buffer (CSK), revealed co-localization between ER-mCherry-LacI-FokI-DD and 53BP1 (Figure 3B). However, upon depletion of endogenous KDM2A, and consistent with the 53BP1 IRIF data (Figure 3A), the recruitment of 53BP1 to DSBs induced within the LacO array was lost (Figure 3B). Thus, two independent approaches revealed that KDM2A depletion impairs the recruitment of 53BP1 to DSBs.

To further understand the role of KDM2A in the DNA damage response we evaluated its role in entry into and exit from the G2/M checkpoint upon IR by monitoring the levels of a mitotic marker (phosphorylation of Histone H3 on Serine 10, termed H3-S10ph). Importantly, prior to irradiation, control and KDM2A-depleted cells showed similar cell cycle profiles (Supplementary Figure 3). Entry into the G2/M checkpoint was unaffected by depletion of KDM2A as these cells entered the checkpoint with similar kinetics to control cells (Figure 3C). However, relative to control cells, KDM2A depleted cells exited the G2/M checkpoint between 3 and 4 hours earlier than control cells. This observation indicated that irradiation of cells depleted of KDM2A results in faster progression into mitosis without proper repair of damaged DNA. Noteworthy, immunoblotting performed with cell lysates obtained from different time points following irradiation did not reveal any striking difference in ATM phosphorylation in cells depleted of KDM2A relative to control cells (Supplementary Figure 4). This observation suggests that KDM2A depletion does not significantly impair checkpoint activation (and see also Figure 3C). However, in support of our findings, depletion of KDM2A has been shown to increase the sensitivity of breast cancer cells to cisplatin-induced DNA damage [33].

### The demethylase activity and zinc finger domain of KDM2A mediate its accumulation at DSBs and are required for the recruitment of 53BP1 to DNA breaks

**Figure 4:**
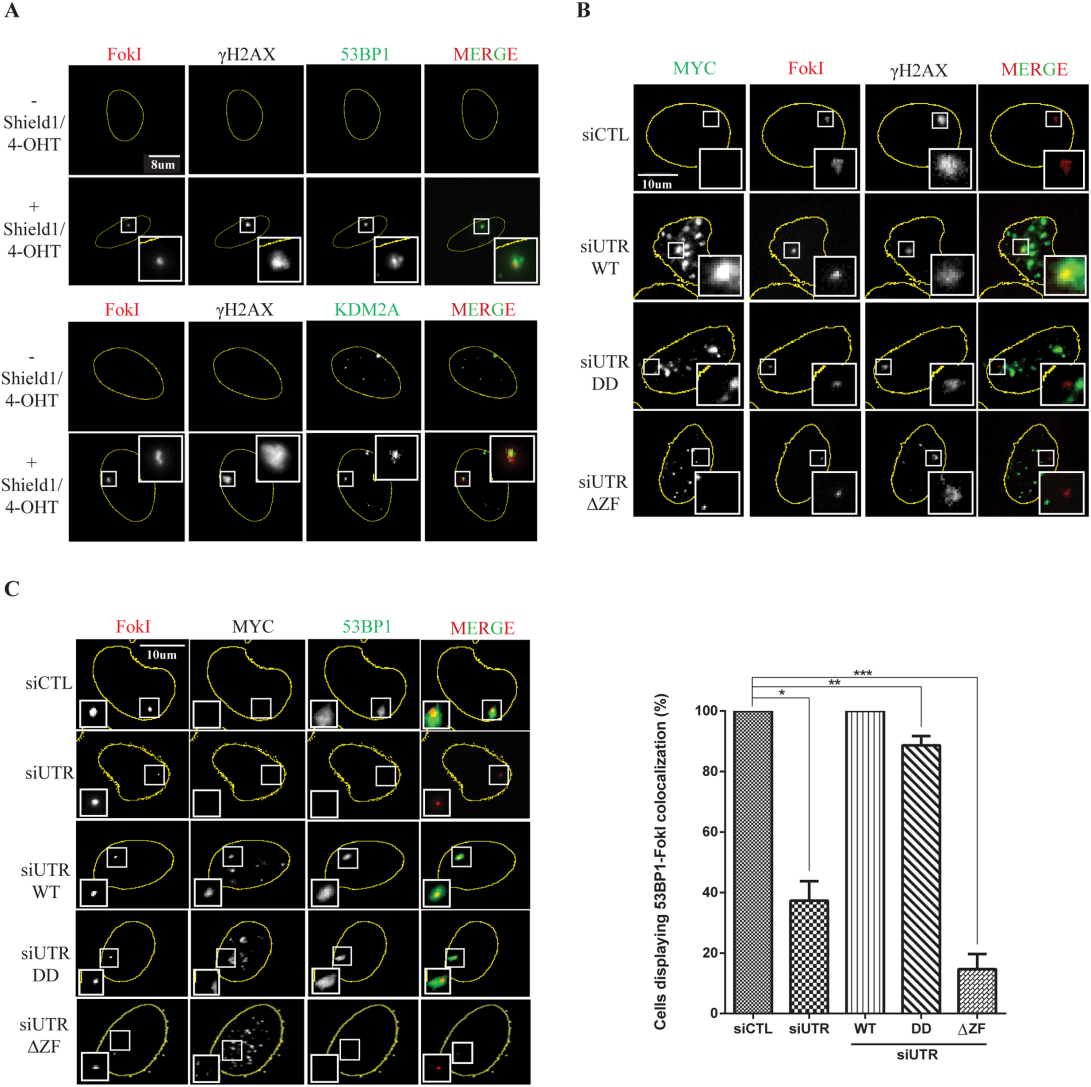
Recruitment of KDM2A and 53BP1 to DSBs depends on its demethylase activity and intact zinc finger domain **(A)** Endogenous KDM2A is recruited to DSBs. Detectable KDM2A at the FokI-induced breaks upon CSK treatment. U2OS-DSB-reporter cells were treated or not with Shield-1 and 4-OHT to induce expression of ER-mCherry-LacI-FokI-DD (FokI) (red). Cells were immunostained with anti-γH2AX, anti-53BP1 (green, upper panel) and anti-KDM2A (green, lower panel). Immunofluorescence analyses were conducted using cells that were pre-extracted with CSK buffer. **(B)** The demethylase activity and zinc finger domain of KDM2A are necessary for its recruitment to DSBs. U2OS-DSB-reporter cells were transfected with siCTL or siUTR and plasmids encoding for MYC-tagged variants of KDM2A. Following transfection, cells were treated with Shield-1 and 4-OHT to induce expression of ER-mCherry-LacI-FokI-DD (FokI) (red). Immunofluorescence analyses were conducted with anti-MYC (green) and anti-γH2AX. **(C)** Focal accumulation of 53BP1 at DSBs is modulated by the demethylase activity and zinc finger domain of KDM2A. U2OS-DSB-reporter cells were transfected with siCTL or siUTR and plasmids encoding for MYC-tagged variants of KDM2A. Cells were treated with Shield-1 and 4-OHT to induce expression of ER-mCherry-LacI-FokI-DD (FokI) (red). Immunofluorescence analyses were conducted with anti-MYC and anti-53BP1 (green). The percentage of cells displaying colocalization between 53BP1 and FokI fusion protein was quantified in each condition (right panel). Experiments were replicated four times. Single (^*^), double (^**^) and triple (^***^) asterisks indicate p<0.05, two-tailed paired Student t-test. Error bars indicate standard deviation.

Although KDM2A forms focal structures in undamaged cells that are sensitive to treatment with CSK buffer, recruitment of KDM2A into IRIF was not detectable (Supplementary Figure 5). We performed immunofluorescence analyses in the U2OS-DSB-reporter cell line using antibodies against 53BP1, γH2AX and KDM2A (Figure 4A). Induction of DSBs in U2OS-DSB-reporter cells resulted in 53BP1 and γH2AX focal accumulation at DSBs induced within the LacO array (Figure 4A, top panel). Interestingly, the signal corresponding to ER-mCherry-LacI-FokI-DD was less diffuse than the signal obtained for either γH2AX or 53BP1 consistent with the fusion endonuclease being located directly at the LacO array rather than the surrounding chromatin. Immunofluorescence analyses using anti-KDM2A revealed that KDM2A also co-localised with the ER-mCherry-LacI-FokI-DD nuclease at DSBs induced within the LacO array but within the signal corresponding to γH2AX modified chromatin (Figure 4A, lower panel). Notably, pre-extraction with cytoskeletal buffer to remove nucleoplasmic and weakly chromatin-bound KDM2A, was required for the visualization of KDM2A localized at DSBs within the LacO array.

Immunofluorescence performed without CSK pre-extraction revealed a pan-nuclear distribution of KDM2A with numerous spots that did not change significantly upon Shield-1 and 4-OHT treatment (Supplementary Figure 1C and also Figure 3B).

Next, we used the U2OS-DSB-reporter system to assess the importance of the lysine demethylase activity and zinc finger domain of KDM2A in mediating its recruitment to DSBs. Endogenous KDM2A was depleted in U2OS-DSB-reporter cells that were also transfected with an empty plasmid or plasmids encoding MYC-tagged (MYC-KDM2A-WT), demethylase deficient (KDM2A-DD) or zinc finger deleted (KDM2A-ΔZF). Immunofluorescence was performed with U2OS-DSB-reporter cells in which DSBs were induced by addition of Shield-1 and 4-OHT and pre-extracted with CSK (Figure 4B). Similar to endogenous KDM2A (Figure 4A, lower panel), MYC-tagged wild type KDM2A was recruited to nuclease-induced DSBs as evidenced by co-localisation between MYC-KDM2A-WT and ER-mCherry-LacI-FokI-DD (Figure 4B). Importantly, unlike WT, the MYC-KDM2A-DD and MYC-KDM2A-ΔZF mutants failed to accumulate at DSBs. In agreement with Tanaka *et al.*[34], we observed that MYC-tagged wild-type KDM2A appeared to localise within nucleoli in the absence of DNA damage (Supplementary Figure 6). Similarly to WT KDM2A, the demethylase-deficient and zinc finger deletion mutants of KDM2A also localized within nucleoli. However, after treatment with ionizing radiation, rather than localising within nucleoli, WT KDM2A either localised to the nucleolar periphery or appeared to be excluded from nucleoli. Interestingly, the zinc finger deletion mutant of KDM2A remained localised to the nucleoli, albeit more diffusely than WT, while the demethylase-deficient KDM2A behaved more similarly to the WT.

As previously demonstrated in Figure 3A-3B, depletion of endogenous KDM2A resulted in impaired recruitment of 53BP1 to DSBs. Moreover, Figure 4B shows that KDM2A itself is recruited to DSBs in a process that depends on its demethylase and zinc finger domain. Consequently, we next evaluated the ability of the KDM2A mutants MYC-KDM2A-DD and MYC-KDM2A-ΔZF to regulate the accumulation of 53BP1 at DNA breaks. Reintroduction of wild type MYC-KDM2A into U2OS-DSB-reporter cells depleted of endogenous KDM2A allowed normal co-localization between 53BP1 and the DSBs induced within the LacO array (Figure 4C). However, the percentage of cells displaying co-localization between 53BP1 and the fusion endonuclease was significantly reduced upon expression of MYC-KDM2A-DD or MYC-KDM2A-ΔZF. In line with the 53BP1 IRIF data (Figure 3A), deletion of the zinc finger domain of KDM2A had a more drastic effect on preventing 53BP1 recruitment to induced DSBs.

Interestingly, within the population of cells displaying efficient siRNA-mediated depletion of KDM2A, we observed that the majority (87%) of the cells displayed defective 53BP1 recruitment to Fok1-generated DSBs in the U2OS-DSB reporter cell line, while 13% of the KDM2A-depleted cells still displayed residual 53BP1 recruitment. This likely reflects different extents of KDM2A-depletion within the treated population. (Supplementary Figure 7).

In summary, our data are consistent with the lysine demethylase and zinc finger domains of KDM2A being necessary for the recruitment of KDM2A to DSBs where it is required for efficient 53BP1 focal accumulation at sites of DNA damage.

### The zinc finger motif of KDM2A is required for genome stability by preventing both the accumulation of micronuclei and 53BP1 foci in mitotic chromosomes upon ionizing radiation

**Figure 5:**
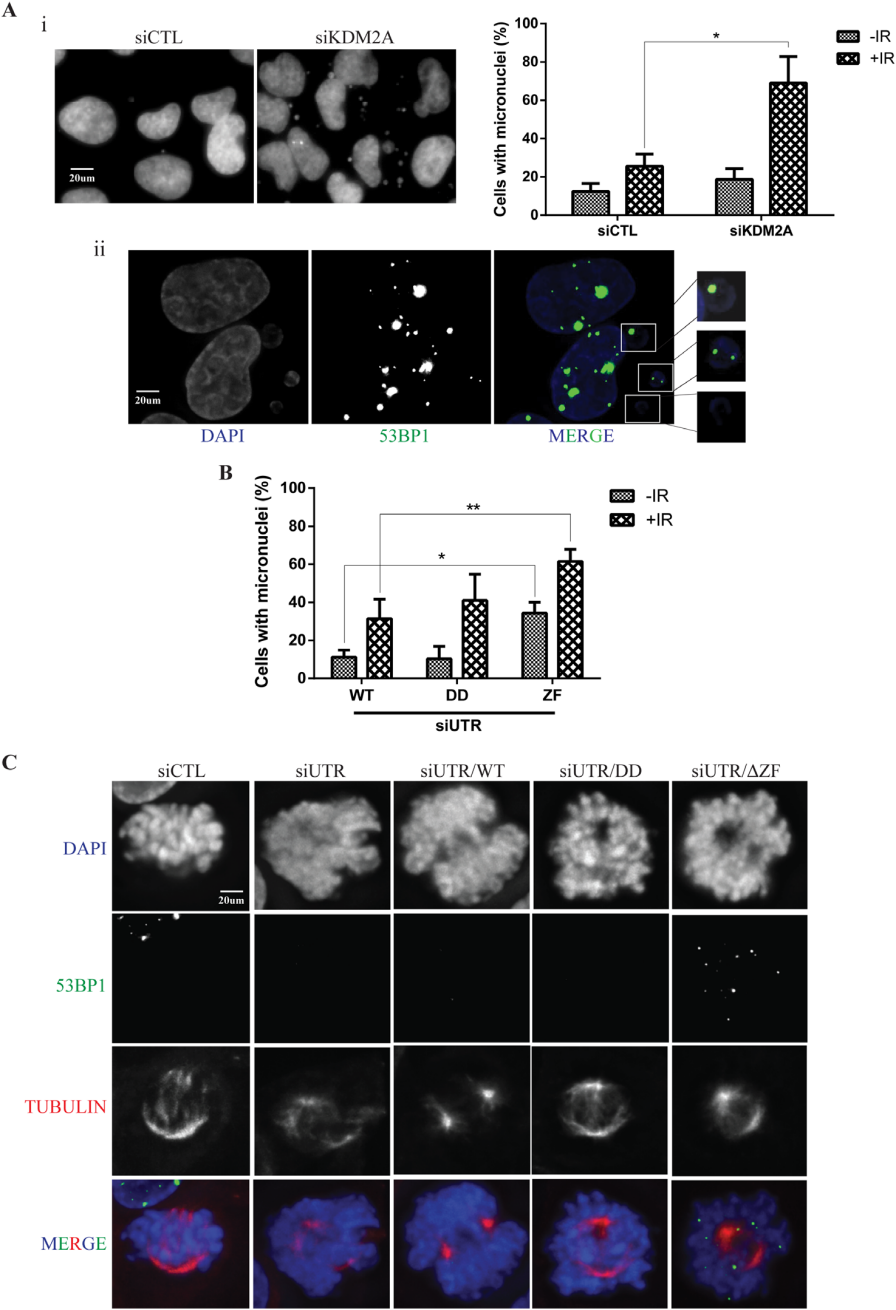
KDM2A modulates genome stability upon IR **(A-i)** KDM2A knockdown sensitizes cells to IR leading to micronuclei formation. U2OS cells transfected with siCTL or siKDM2A were irradiated (2Gy) and fixed 24 hrs later. Cells were stained with DAPI and micronuclei observed by fluorescence microscopy. The percentage of cells harboring micronuclei before and after IR was quantified in each condition (right panel). Experiments were performed in triplicates. Asterisk indicates p<0.05, two-tailed paired Student t-test. Error bars indicate standard deviation. (**A-ii**) 53BP1 forms foci in micronuclei found in KDM2A knockdown cells. Cells transfected with siKDM2A were irradiated (2Gy) and, 24 hrs later, subjected to immunofluorescence with anti-53BP1(green) and DAPI (blue). **(B)** The zinc finger domain of KDM2A is crucial for the maintenance of genome stability. Endogenous KDM2A was downregulated in U2OS cells stably expressing wild type MYC-tagged KDM2A-WT, -DD or -ΔZF mutants by transfection with siUTR. Cells were irradiated (2Gy) or left untreated and the percentage of cells showing micronuclei was determined 24 hrs after IR treatment. Single (^*^) and double (^**^) asterisks indicate p<0.05, two-tailed paired Student t-test. Error bars indicate standard deviation. Experiments were performed in triplicates. **(C)** 53BP1 localization in mitotic cells upon IR. U2OS cells transfected with siCTL or siUTR and, stably expressing or not, wild type MYC-KDM2A (WT), DD or ΔZF mutants were irradiated (2Gy) and fixed 24 hrs later. Cells were subjected to immunofluorescence with anti-53BP1 (green), anti-Tubulin (red) and DAPI (blue).

Defective repair of DNA double strand breaks is known to generate acentric chromosomes and chromatid fragments that in turn give rise to micronuclei [35]. Interestingly, and consistent with premature exit from the IR-induced G2/M checkpoint (Figure 3C), depletion of KDM2A resulted in a significant increase in the number of cells harbouring micronuclei 24 hrs after irradiation (Figure 5Ai). Furthermore, we observed micronuclei displaying 53BP1 foci in KDM2A-depleted cells, suggesting that these structures indeed contained unrepaired DNA (Figure 5Aii).

As previously shown (Figure 2B), KDM2A-DD displayed reduced ability to stimulate 53BP1 ubiquitination while KDM2A-ΔZF was incapable of promoting conjugation of ubiquitin to 53BP1.

Furthermore, cells expressing KDM2A-DD or MYC-KDM2A-ΔZF also displayed defective recruitment of 53BP1 to DSBs (Figure 3A and 4C). Thus, we evaluated the role of these mutants with respect to genome stability using a micronuclei assay. Endogenous KDM2A was depleted in U2OS cells stably overexpressing MYC-tagged KDM2A-WT, KDM2A-DD or MYC-KDM2A-ΔZF and micronuclei were quantified by immunofluorescence (Figure 5B). Unirradiated cells expressing exogenous MYC-KDM2A-WT or MYC-KDM2A-DD displayed similar percentages of cells with micronuclei to the percentage observed upon depletion of endogenous KDM2A (compare Figure 5B with Figure 5Ai, right panel). However, expression of MYC-KDM2A-ΔZF with concomitant depletion of endogenous KDM2A resulted in a significant increase in the percentage of cells harbouring micronuclei both in the absence and after IR treatment (Figure 5B). Interestingly, irradiation promoted a further increase in micronuclei frequency.

While monitoring micronuclei in these experiments, we noticed that mitotic cells expressing MYC-KDM2A-ΔZF displayed numerous 53BP1 foci within condensed mitotic chromatin 24hrs after irradiation (Figure 5C). This suggests the possibility that KDM2A downregulation could result in the recruitment of 53BP1 to chromatin during mitosis using a mechanism dependent upon its zinc finger domain. Interestingly, 53BP1 phosphorylation has been shown to modulate its recruitment to DNA breaks in mitotic cells [36, 37]. Intriguingly, depletion of endogenous KDM2A alone was not sufficient to produce this phenotype.

## DISCUSSION

Thus far, lysine demethylation of both histone [24] and non-histone proteins [38] has been assigned as the primary function of KDM2A. However, KDM2A has also been shown to play additional biological roles such as regulation of β-catenin stability by promoting its ubiquitination [16]. Similarly, KDM2B, the closest homolog of KDM2A, has also been demonstrated to stimulate protein ubiquitination [17]. Thus, emerging evidences indicate that KDM2A executes some of its functions via mechanisms beyond its lysine demethylase activity.

Consistent with these novel findings, we report that KDM2A plays an important role in the mechanisms involved in DNA damage repair and maintenance of genome stability. Our data demonstrates that KDM2A interacts with 53BP1 via a process predominantly mediated by the zinc finger domain of KDM2A, which is reminiscent of the reported association between RAD18 and 53BP1 [14]. This finding suggests that associations between 53BP1 and its molecular partners are frequently mediated by zinc finger domains. Moreover, we observed that amino acid residues spanning positions 1052-1302 of 53BP1 are necessary for its interaction with KDM2A. Notably, these residues have already been shown to play a role in the recruitment of 53BP1 to DNA breaks [26].

Our data indicate, for the first time, that KDM2A stimulates ubiquitination and regulates the stability of 53BP1. While demethylase-deficient KDM2A showed reduced ability to promote 53BP1 ubiquitination, deletion of the zinc finger domain of KDM2A abrogated its capacity to stimulate conjugation of Ubiquitin to 53BP1. Notably, we provide data demonstrating that both endogenous and exogenously-expressed KDM2A are recruited to DSBs. Further analyses of KDM2A mutants revealed that its zinc finger domain and demethylase activity are both necessary for the accumulation of KDM2A at DNA double strand breaks. Interestingly, other lysine demethylases have been shown to accumulate at DNA damage sites [39–43] consistent with enzymes involved in lysine demethylation playing active and important role in the process of DNA repair.

In addition, we demonstrate that the association between KDM2A and 53BP1 is necessary for the proper recruitment of 53BP1 to sites of DNA double strand breaks generated either by ionizing radiation or by the endonuclease activity of FokI. As expected, depletion of endogenous KDM2A followed by complementation with wild type MYC-tagged KDM2A resulted in normal 53BP1 localization to DSBs. However, complementation with demethylase-deficient or zinc finger deleted KDM2A mutants revealed a significant reduction in the recruitment of 53BP1 to DNA double strand breaks. Nevertheless, cells expressing the KDM2A-ΔZF mutant displayed a more severe defect in 53BP1 recruitment to DSBs. Given that this mutant is also unable to stimulate ubiquitination of 53BP1, our findings suggest that lack of KDM2A-driven ubiquitination of 53BP1 prevents its accumulation at DSBs perhaps by affecting the dynamics of processes involved in the recruitment of 53BP1 to DNA breaks. Such processes include a distinctive type of ubiquitination mediated by RNF168 which has been shown to actively modulate the focal accumulation of 53BP1 at DSBs [44]. Alternatively, defective demethylation or ubiquitination of another KDM2A target, rather than 53BP1, could exert an indirect negative effect on the recruitment of 53BP1 to DSBs.

The recruitment of 53BP1 to DSBs is known to be a rapid process. RNF168-mediated ubiquitination of 53BP1 has been shown to promote the initial recruitment to DNA double strand breaks [44]. Of note, 53BP1 degradation observed upon KDM2A expression was mild and exhibited slow kinetics. Thus, similarly to RNF168, we propose that KDM2A-stimulated ubiquitination of 53BP1 could be necessary for the initial recruitment of 53BP1 to breaks and, eventually, a small portion of ubiquitinated 53BP1 would be subjected to degradation. Alternatively, we hypothesize that KDM2A mostly stimulates the attachment of ubiquitin chains on 53BP1 (e.g. K63 linkages) that do not necessarily result in protein degradation.

Overexpression of KDM2A was linked to reduced accumulation of MRE11 at DNA breaks leading to a potential decrease in DNA repair [20, 45]. However, recruitment of 53BP1 upon overexpression of KDM2A was not evaluated in this study [20, 45]. Our data demonstrates that depletion of KDM2A, in contrast to its overexpression, prevents the recruitment of 53BP1 to DSBs. In support of our findings, 53BP1 focal recruitment to DNA double strand breaks has been reported by at least two other studies to occur independently of MRE11 [46, 47]. In further support of our data, KDM2A depletion was shown to increase the sensitivity of breast cancer cells to cisplatin-induced DNA damage [33].

A previous study by Frescas *et al* [3] reported that KDM2A depletion reduces mitotic fidelity and genome stability due to augmented H3K36me2 levels at pericentric satellite repeats. In contrast, Bergmann *et al.* showed more recently that elevated levels of H3K36me2 at centromeric chromatin are actually necessary for chromosome stability [48]. Thus, the link between KDM2A depletion and reduced genome stability proposed by Frescas *et al* [3] is unlikely to be a result of alterations in H3K36me2 levels at pericentric satellite repeats. We note that Frescas *et al* [3] did not perform complementation experiments using KDM2A-depleted cells expressing wild type or KDM2A mutants as a strategy to establish a clear connection between KDM2A-regulated expression of pericentric repeats and mitotic fidelity.

Indeed, our data confirm that depletion of KDM2A leads to genome instability [3, 6]. Nevertheless, we observed that this decreased genomic stability due to KDM2A depletion is directly related to impaired recruitment of 53BP1 to DSBs and exasperated upon DNA damage. In addition, following irradiation, KDM2A depleted cells displayed premature entry into mitosis upon exit from the G2/M checkpoint, suggesting that KDM2A depletion results in progress into mitosis with unrepaired DSBs. Of note, unrepaired double-stranded DNA breaks that arise from defects in the NHEJ pathway are known to be a significant source of micronuclei formation [35]. Therefore, we propose that the genome instability observed in KDM2A-depleted cells, which is exacerbated upon IR, is due to defective 53BP1 recruitment to DSBs and not a result of elevated levels of H3K36me2 at centromeric as proposed by Frescas *et al* [3].

Specific phosphorylation of 53BP1 was shown to prevent its recruitment to DSBs during mitosis [36, 37]. Expression of 53BP1 that cannot be phosphorylated on residues threonine 1609 and serine 1618 (T1609A/S1618A) results in 53BP1 recruitment to DNA breaks in mitotic cells and genomic instability [36]. Moreover, Orthwein *et al.*[37] showed that, during mitosis, 53BP1-T1609A/S1618A can promote DSB repair that ultimately results in telomere fusions. Interestingly, our findings show that cells concomitantly depleted of endogenous KDM2A and expressing KDM2A-ΔZF displayed 53BP1 focal accumulation on metaphase chromosomes observed in cells undergoing mitosis 24hrs after irradiation. It remains unknown whether these 53BP1 foci correspond to DNA breaks or to another structure. Of note, these regions of 53BP1 focal accumulation were not observed in mitotic cells expressing wild type or demethylase-deficient KDM2A.

This observation suggests that expression of the zinc finger deletion mutant KDM2A may affect the phosphorylation status of 53BP1 on residues threonine 1609 and serine 1618 in mitotic cells. Therefore, the incidence of micronuclei observed in cells expressing KDM2A-ΔZF may be directly related to the occurrence of telomere fusions. In fact, the high incidence of chromosome bridges reported upon KDM2A depletion [3] suggests that these structures potentially originate from telomere to telomere end fusions.

Altogether, we report several novel findings: KDM2A associates with 53BP1 and modulates its ubiquitination; KDM2A depletion leads to defective recruitment of 53BP1 to DSBs; KDM2A itself is recruited to DNA double strand breaks through a mechanism that requires an intact zinc finger domain and demethylase activity; deletion of the KDM2A zinc finger domain disrupts its capacity to associate and ubiquitinate 53BP1 which leads to increased genomic instability and results in 53BP1 focal accumulation in mitotic cells following irradiation.

## MATERIALS AND METHODS

### Plasmids and siRNA

The plasmid pLPC-Puro-FLAGKDM2AWT was constructed by cloning PCR-amplified FLAG-tagged KDM2A from pcDNA3FLAGKDM2A (a gift from Dr. Yi Zhang, Harvard Medical School) into pLPC-Puro. A MYC-tagged KDM2A vector was created by PCR amplification of KDM2A from pLPC-Puro-FLAGKDM2AWT and cloning into pLPC-Puro-MYC. A demethylase -deficient KDM2A mutant, pLPC-Puro-MYC-KDM2ADD, was created by site-directed mutagenesis of pLPC-Puro-MYC-KDM2AWT. This mutant contains the histidine and aspartic acid residues at position 212 and 214 mutated to alanine. A plasmid encoding for the MYC-tagged KDM2A mutant lacking the zinc finger domain (pLPC-Puro-MYCKDM2AΔZF) was obtained by deletion of the amino acid residues spanning positions 564-610 of pLPC-Puro-MYCKDM2AWT. The plasmid pRK5-HA-Ubiquitin-WT was a gift from Ted Dawson (Addgene plasmid # 17608) [49] and pcDNA5-FRT/T0-Flag-53BP1 was a gift from Daniel Durocher (Addgene plasmid # 52507) [50]. Plasmids encoding for HA-tagged 53BP1 wild type (pCMH6K53BP1WT) and mutants (pCMH6K53BP115A/Q, pCMH6K53BP11-1051, pCMH6K53BP1Δ1-1051, pCMH6K53BP1Δ1052-1302, pCMH6K53BP1ΔTudor and pCMH6K53BP1ΔBRCT were a gift from Dr. Michael Huen (The University of Hong Kong) and Dr. Junjie Chen (The University of Texas MD Anderson Cancer Center). This plasmids have been previously described [26].

### Antibodies

The following primary antibodies were used in this study: anti-53BP1 Novus Biologicals (NB100-904), anti-Tubulin (B512 clone) Sigma, anti-MYC 9E10 clone, anti-HA 12CA5 clone, anti-FLAG M2 Sigma (F3165), anti-H3pSer10 Millipore (06-570), anti-pan methyl lysine Abcam (ab7315), anti-JHDM1A/KDM2A Bethyl (A301-475A), anti-phospho H2AX Ser139 (γH2AX) Abcam (ab2893), anti-MYC Bethyl (A190-105A), anti-ubiquitinylated proteins FK2 Millipore (04-263) and anti-phospho-ATM Ser1981 R&D Systems (AF1655). Fluorescent-labeled secondary antibodies conjugated to FITC or TRITC were obtained from Jackson ImmunoResearch.

### Cell culture and stable cell lines

Human osteosarcoma (U2OS) and human embryonic kidney (HEK293T) cell lines were purchased from LGC Standards, UK. U2OS-DSB reporter cell line was a gift from Dr. Roger A. Greenberg (University of Pennsylvania, Philadelphia). These cells were cultured in Dulbecco’s modified Eagle high glucose medium (Lonza) supplemented with 10% fetal bovine serum (FBS) (Lonza) and 1% Penicillin-Streptomycin (Sigma). U2OS and HEK293T cells stably expressing MYC-tagged KDM2A were generated by transfection of pLPC-Puro-FLAGKDM2AWT, pLPC-Puro-FLAGKDM2ADD or pLPC-Puro-FLAGKDM2AΔZF and further selection with Puromycin (2μg/ml) for 15 days. Control cell lines were generated by transfection of pLPC-Puro-MYC. All plasmid transfections were performed with Lipofectamine 2000 (Thermo Fisher).

### Immunofluorescence

U2OS stable cell lines expressing or not MYC-tagged KDM2A constructs were transfected with 40 nM of siRNA targeting GFP (siCTL) or siKDM2A-UTR targeting the 3’-UTR region of the KDM2A mRNA. This siRNA was used to downregulate the expression of endogenous KDM2A but not exogenously expressed MYC-tagged KDM2A variants. Forty-eight hours after transfection, cells were gamma-irradiated (IR) with 2Gy and harvested 1h and 24hrs later. Cells were then fixed for 10 min at room temperature (RT) in 4% Paraformaldehyde (PFA), permeabilized for 2 min at RT with 0,125% Triton X-100 diluted in phosphate-buffered saline (PBS) and later blocked for 1h at RT in 10% FBS/PBS. Cells were then washed 3 times with 1x PBS and incubated with 5% FBS diluted in PBS containing primary antibody for 1h at RT. Later, cells were washed 3 times with 1x PBS and incubated with fluorescent-labeled secondary antibody diluted in 5% FBS/PBS for 1h at RT. Cellular nuclei was stained with DAPI mounting media (Vectashield).

The sequence of siCTL was GCCACAACGUCUAUAUCAU and siKDM2A-UTR was GUUUGUUUCUUCCAAUCUGCUGAAU. All siRNA transfections were performed with Oligofectamine (Thermo Fisher).

For the analysis of endogenous KDM2A accumulation at DSBs, 1.5×10^5^ U2OS-DSB-reporter cells were plated on 35mm dishes and, 24hrs later, treated or not for 5h with Shield-1 (1uM) and 4OHT (1uM). Cells were then processed for immunofluorescence following the protocol described above. Immunofluorescence analyses of 53BP1 accumulation at DSBs in the presence or not of endogenous KDM2A was conducted by plating 1.5×10^5^ U2OS-DSB reporter cells on 35mm dishes and, 24hrs later, transfecting them with 50uM of siCTL or siKDM2A (UGCUGUAUCUGCAAUGAGA). Forty-eight hours after transfection, cells were treated or not for 5h with Shield-1 (1uM) and 4OHT (1uM) and immunostained with the indicated antibodies.

The recruitment of MYC-tagged wild type KDM2A and mutants to DSBs was evaluated by plating 2×10^5^ U2OS-DSB-reporter cells on 35mm dishes and, 24hrs later, transfecting them with 50uM of siCTL or siKDM2A-UTR. On the next day, cells were transiently transfected with 5 ug of an empty vector (pLPC-Puro) or pLPC-Puro-MYC-KDM2AWT, pLPC-Puro-MYC-KDM2ADD and pLPC-Puro-MYC-KDM2AΔZF. Cells were washed once with 1x PBS and fresh media was added 6 hrs after plasmid DNA transfection. On the next day, cells were treated or not for 5h with Shield-1 (1uM) and 4OHT (1uM) and immunostained with the indicated antibodies. Where mentioned, cells were pre-extracted for 10min at room temperature with CSK buffer (100mM NaCl, 300mM Sucrose, 10mM Pipes pH6.7, 3mM MgCl_2_, 0,2% Triton X-100) before fixation.

### Microscopy and image analysis

Images were acquired with an Olympus IX73 inverted microscope (Imvisage, Imsol system). Each image was taken as Z-stacks (0.5μm thickness) which were then deconvolved with the Huygens software (Scientific Volume Imaging). Quantification of 53BP1 foci was performed with the ImageJ software. The parameters for the 53BP1 foci quantification were set on the positive control (siCTL+IR) and kept constant throughout the entire analysis. Images of 16-bit were converted to 8-bit and split into three channels creating three individual images. The DAPI channel was used to adjust the threshold in order to highlight the nuclei. The FITC channel, corresponding to 53BP1 staining, was used to highlight the 53BP1 foci as single points (black dots on a white background). Equal noise tolerance was then kept constant throughout the images analyzed. The number of foci per cell was then calculated by dividing the raw integrated density by the maximum gray value for each cell.

### G2/M checkpoint assay

U2OS cells were transfected with 40 nM of siCTL or previously validated siKDM2A (UGCUGUAUCUGCAAUGAGA) [16]. After 24 hrs, cells were split and re-plated on 7 new 35mm culture dishes and incubated for another 24 hours. This procedure was followed to ensure that the efficiency of the siRNA transfection was similar across all the time points collected. Cells were then irradiated or not with 2Gy and harvested at specific time points. Staining with anti-H3pSer10 and propidium iodide/RNAse treatment followed by flow cytometry analysis was conducted as previously described [51].

### Immunoblotting and immunoprecipitations

HEK293T cells were lysed in lysis buffer (0.5% Triton-X100, 150 mM NaCl, 50 mM Tris-HCl pH 8.0 and 20% Glycerol) containing protease (Leupeptin, Pepstatin, PMSF) and phosphatase (Na_2_VO_4_, NaF) inhibitors and Benzonase (1:1000) Sigma. Lysates were incubated on ice for 40 min and later cleared by centrifugation at 14,000 rpm for 10 min at 4°C. Supernatant was saved and protein concentration determined by Bradford assay. Proteins were then resolved by SDS-polyacrylamide gel electrophoresis, transferred to a nitrocellulose membrane, incubated with the appropriate antibodies and exposed to X-ray films. For immunoprecipitations, equal amounts of total cell extracts obtained with lysis buffer were used for each sample. Extracts were incubated on ice with primary antibody for 2 hrs. Protein G Sepharose beads (GE healthcare) were then added to lysates which were further incubated for 30 min at 4°C under rotation. Beads were washed 3 times with lysis buffer and proteins bound to beads were eluted in 1x Laemmli sample buffer. Alternatively, immunoprecipitations were conducted with anti-FLAG M2 agarose beads (Sigma).

### Analysis of ATM phosphorylation

Levels of ATM phosphorylation on Serine 1981 were evaluated by transfecting 50uM of siGFP or siKDM2A into 1×10^5^ U2OS cells plated in 35mm dishes. Forty-eight hours later, cells were irradiated with 2Gy and harvested at the indicated timepoints. Subsequently, cells were lysed for 1 hour on ice with 1X lysis buffer containing protease and phosphatase inhibitors and benzonase. Supernatants were recovered after spinning down the samples at 14000 rpm for 10min at 4°C. Protein concentration was determined by Bradford protein assay. A total of 40μg of crude cells extract was resolved by SDS-polyacrylamide gel electrophoresis, transferred to a nitrocellulose membrane and incubated, sequentially, with anti-phospho ATM Ser1981, anti-ATM and anti-KDM2A. Following incubation with HRP-conjugated secondary antibody, the membrane was washed and exposed to X-ray films.

### Cell cycle analysis

U2OS cells were plated 24 hours prior siRNA transfection of GFP and KDM2A. Forty-eight hours after transfection, cells were irradiated with 2Gy and harvested at the indicated timepoints. Cells were washed twice in 1X PBS and then fixed overnight at 4°C in 70% ice-cold ethanol. Subsequently, cells were washed once in 1X PBS and resuspended in 1X PBS containing 40μg/ml of Propidium Iodide and 250μg/ml of RNAse A and incubated for 30 minutes in the dark prior to flow cytometry analysis.

We thank the Flow Cytometry Core Facility, Biomedical Sciences Building (National University of Ireland, NUI Galway).

## SUPPLEMENTARY MATERIALS FIGURES


