# Supplementary material for: Recruitment of lysine demethylase 2A to DNA double strand breaks and its interaction with 53BP1 ensures genome stability
Source: Oncotarget. 2018 Mar 23;9(22):15915–30. doi: 10.18632/oncotarget.24636 (PMC5882307; doi:10.18632/oncotarget.24636)
Supplement: Supplementary file 1 [file oncotarget-09-15915-s001.pdf]

Recruitment of lysine demethylase 2A to DNA double strand breaks and its interaction with 53BP1 ensures genome stability

SUPPLEMENTARY MATERIALS

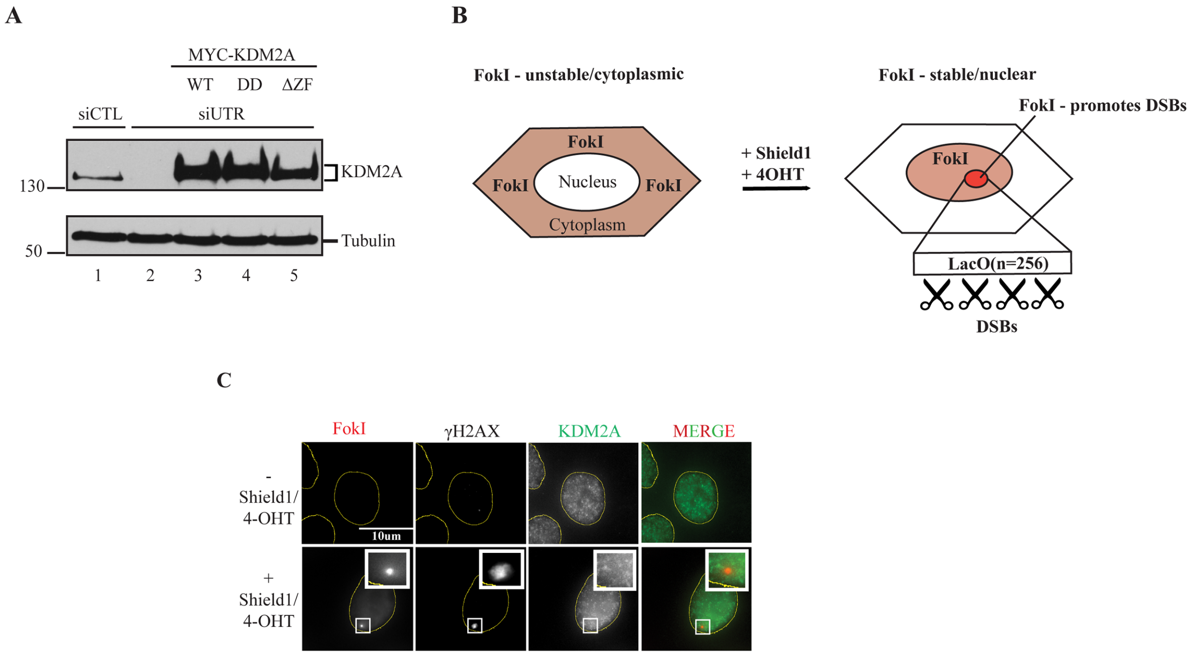

Supplementary Figure 1: (A) U2OS cells stably expressing MYC-tagged KDM2A variants were transfected with siCTL or siUTR. Efficient downregulation of endogenous KDM2A and expression of MYC-tagged KDM2A variants was observed by immunoblotting with anti-KDM2A. Relative loading was shown by immunoblotting with anti-Tubulin. **(B)** Schematic of the U2OS-DSB-reporter cell line. Upon treatment with Shield1 and hydroxytamoxifen (4-OHT), unstable and cytoplasmic ER-mCherry-LacI-FokI-DD (FokI) (light red) is stabilized and translocated to the nucleus where it is recruited to LacO repeats (dark red) and promotes DSBs. **(C)** U2OS-DSB-reporter cells were treated (+) or not (-) with Shield-1 and 4-OHT to induce expression of ER-mCherry-LacI-FokI-DD (FokI) (red). Cells were immunostained with anti-γH2AX and anti-KDM2A (green). Immunofluorescence analyses were conducted with cells that were not pre-extracted with cytoskeleton buffer (CSK).

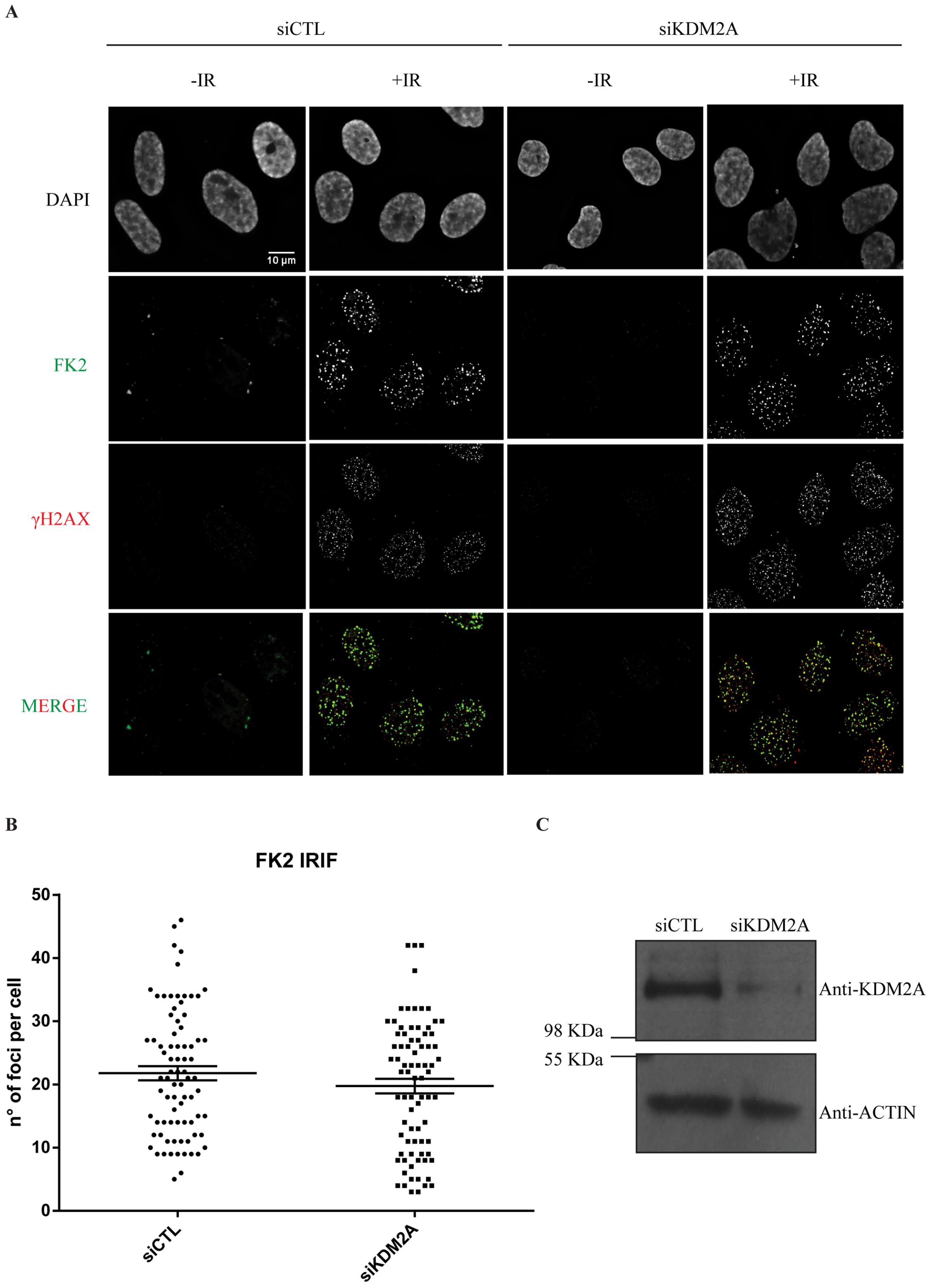

Supplementary Figure 2: (A) Protein ubiquitination upon IR was evaluated inU2OS cells transfected with siCTL or siKDM2A and immunostained with anti-FK2 (green), anti-γH2AX (red) and DAPI. (**B**) Data shown in (A) was quantified. Graph representing the number of FK2 IRIF in cells transfected with the indicated siRNA. (**C**) KDM2A knockdown efficiency in cells shown in (A) was determined by immunoblotting of total cell extracts with anti-KDM2A and anti-Actin.

A

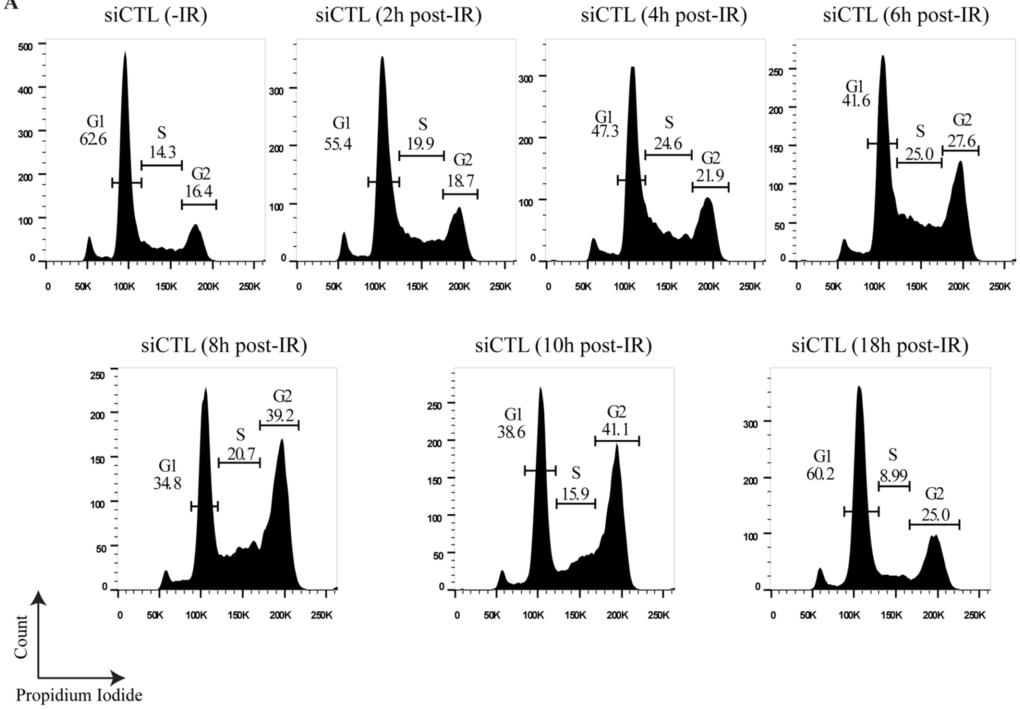

B

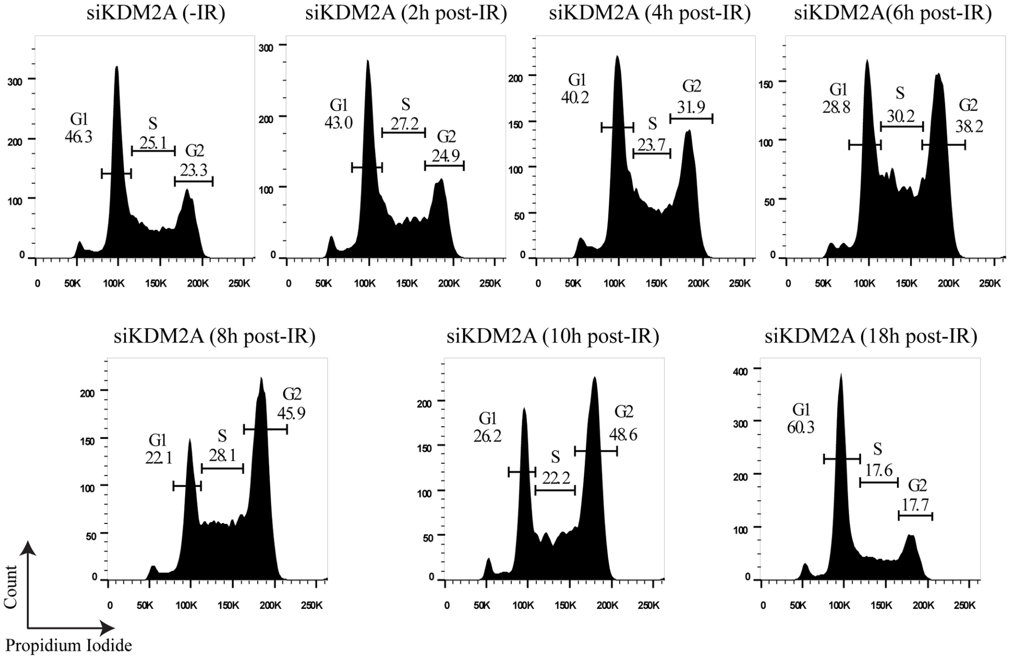

Supplementary Figure 3: **(A)** Flow cytometry analyses demonstrating the cell cycle profile of U2OS cells transfected with siCTL. Cells were collected at the indicated timepoints after irradiation and stained with propidium iodide in the presence of RNAse. **(B)** Cells transfected with siKDM2A were analyzed as described in (A).

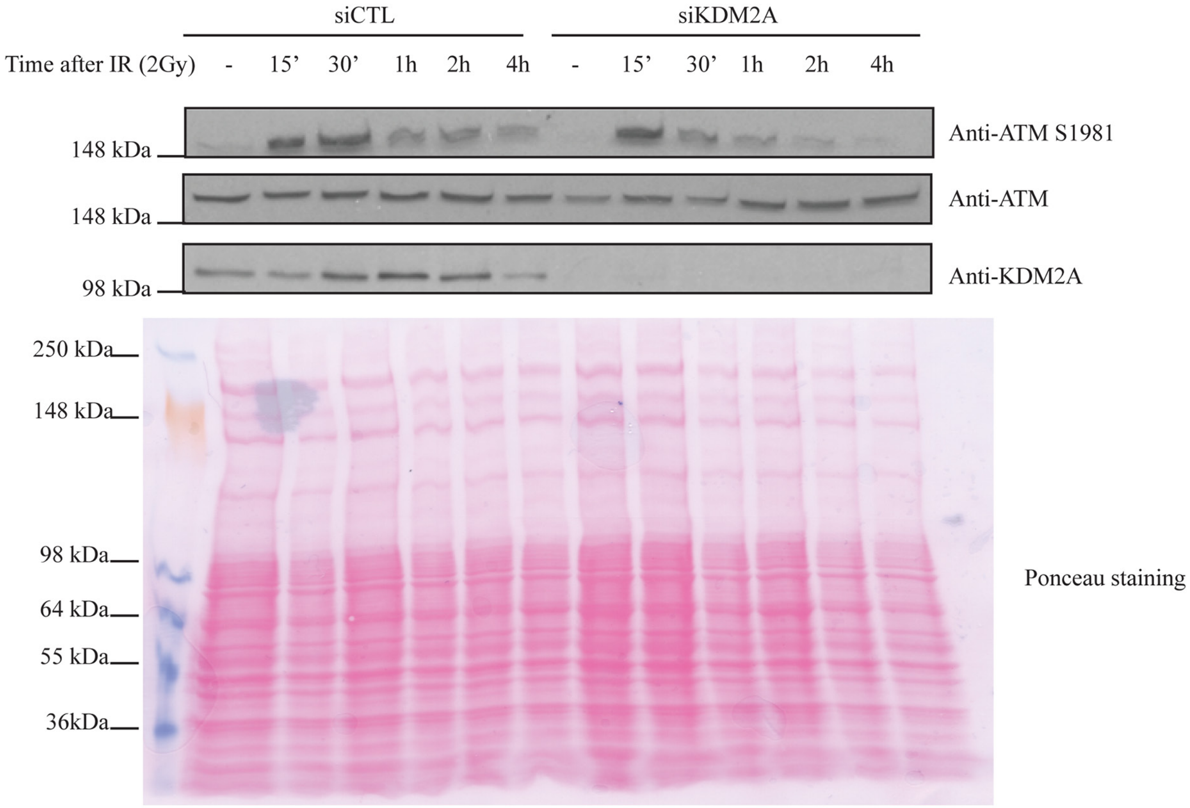

Supplementary Figure 4: ATM phosphorylation at the specified timepoints following irradiation was assessed by immunoblotting total cell lysates from siCTL- and siKDM2A-transfected U2OS cells with anti-ATM, anti-ATM S1981 and anti-KDM2A.

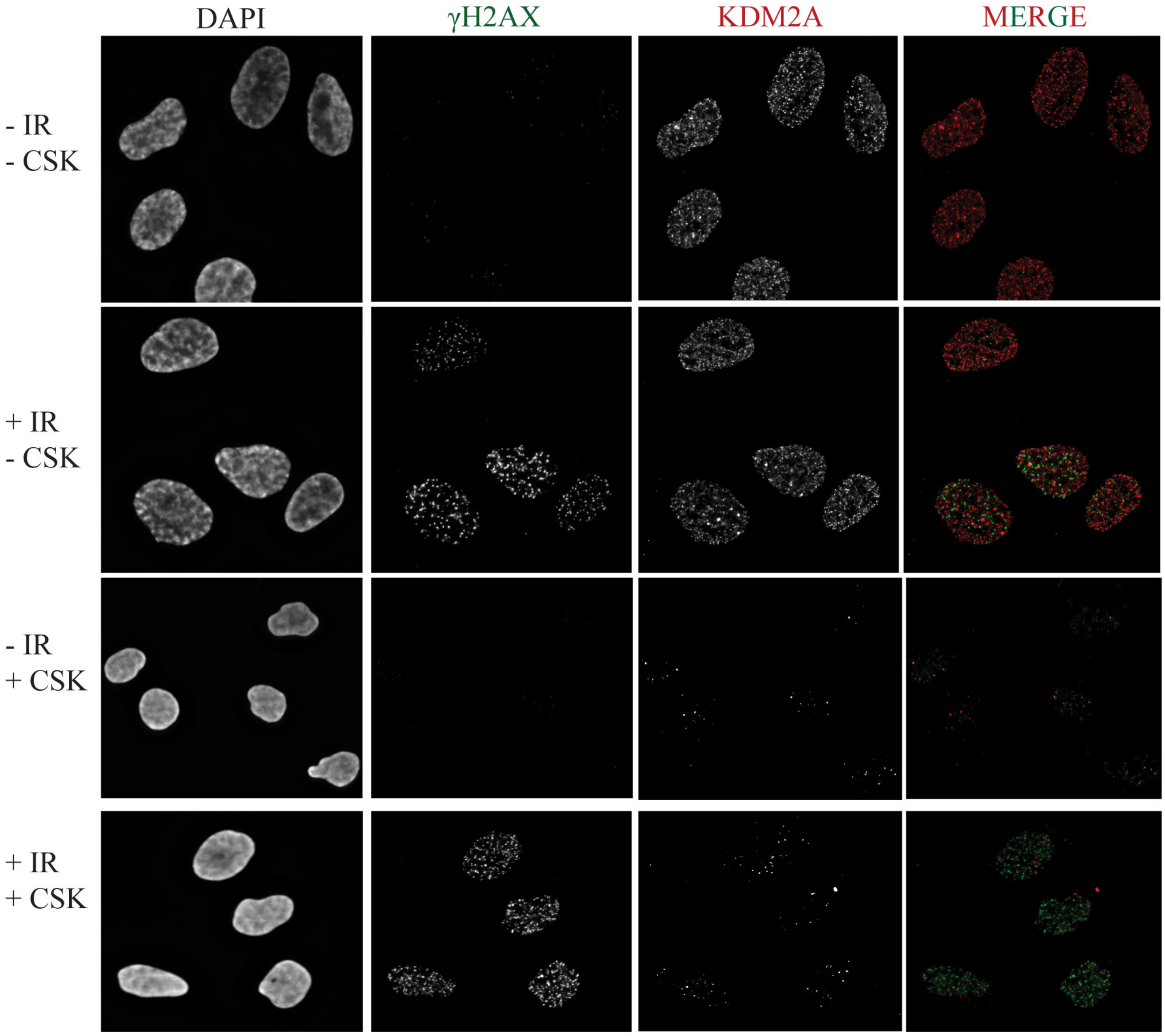

Supplementary Figure 5: KDM2A does not colocalize with γH2AX IRIF. U2OS cells irradiated or not, were immunostained with anti-γH2AX (green), anti-KDM2A (red) and DAPI in the absence or presence of CSK buffer.

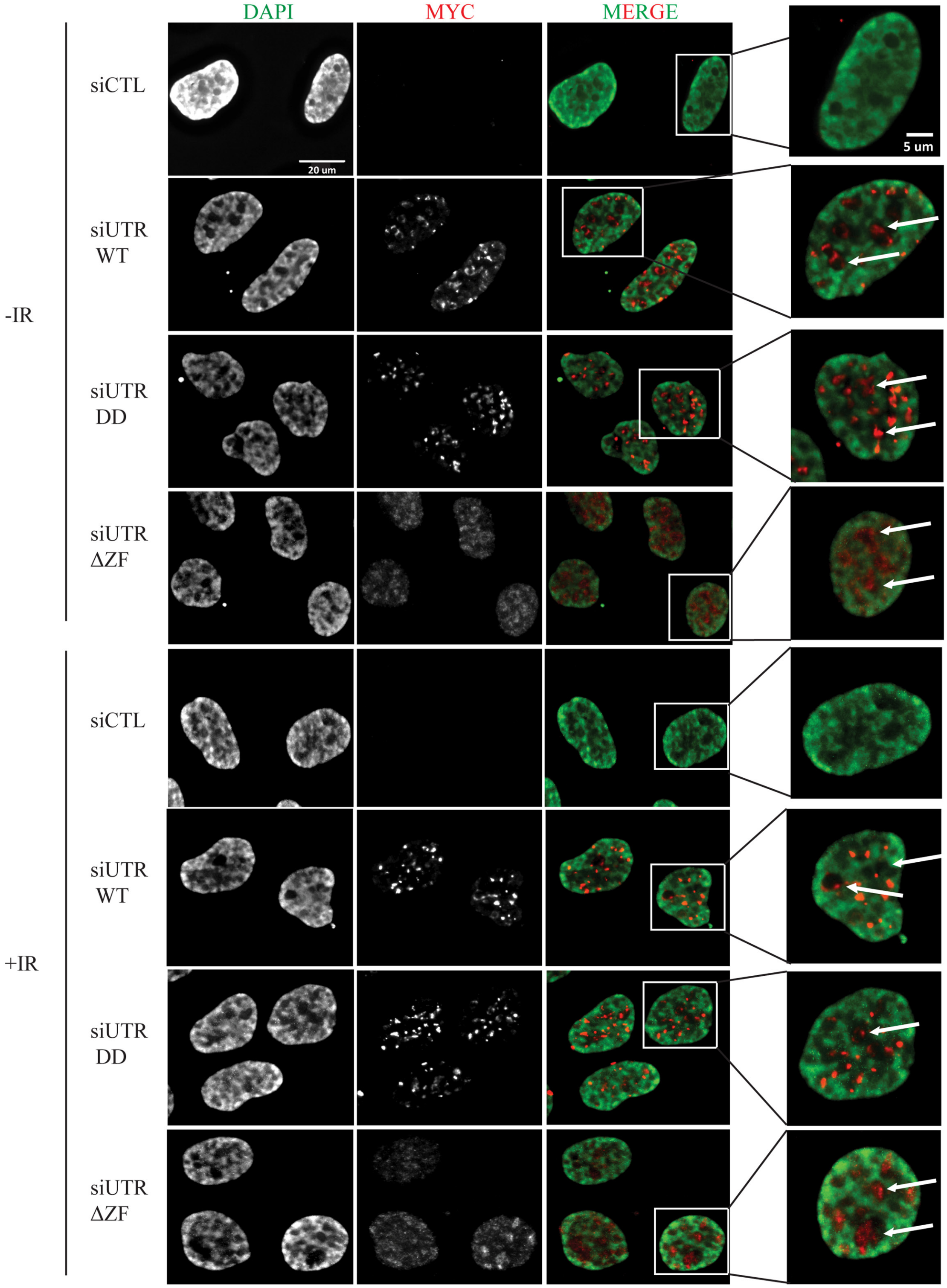

Supplementary Figure 6: Nucleolar localization of KDM2A variants in irradiated and non-irradiated cells. U2OS cells were immunostained with anti-MYC (red) and DAPI (green). Nucleoli were identified by lack of DAPI staining. White arrows indicate KDM2A localization within the nucleolus.

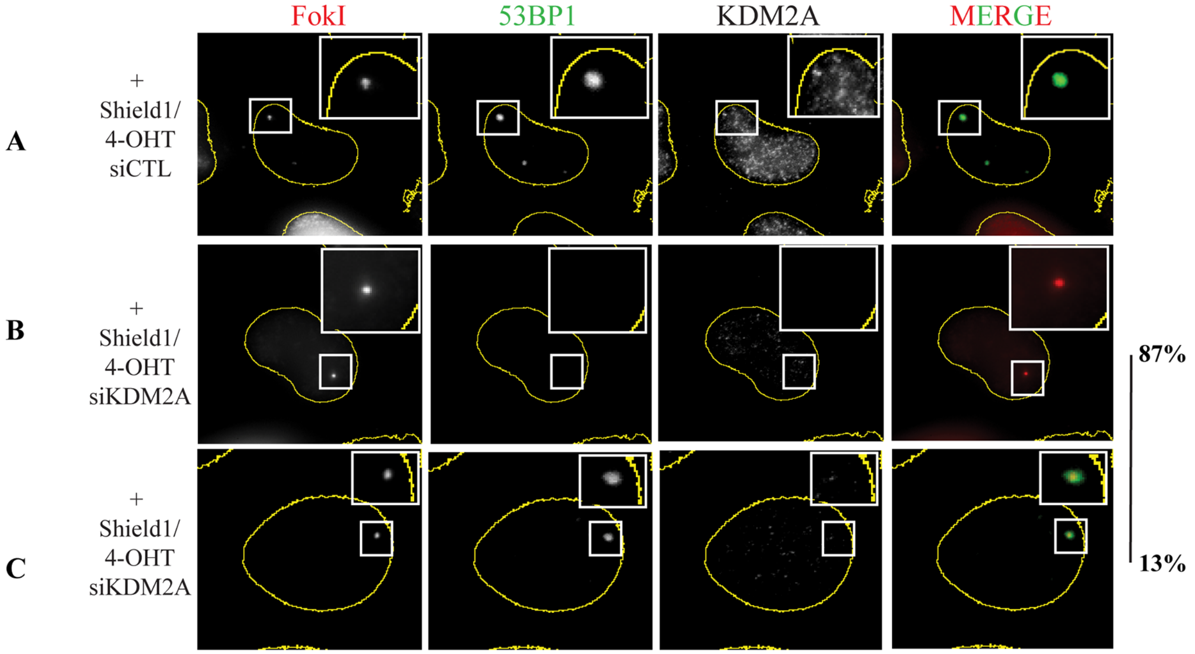

Supplementary Figure 7: Colocalization between FokI and 53BP1 in U2OS-DSB-reporter cells transfected with siCTL or siKDM2A. The efficiency of KDM2A silencing via siRNA transfection plays a partial role in 53BP1 recruitment to DSBs. Immunostaining analyses of cells transfected with siCTL showed colocalization between FokI (red) and 53BP1 (green) **(A)**. The cell population displaying effective depletion of KDM2A via siKDM2A transfection revealed that 53BP1 still colocalized with FokI in 13% of these cells (compare panel **B** with **C**).
